# Arginine-Modified 3D-Printed Chromatographic Supports

**DOI:** 10.3390/pharmaceutics14112266

**Published:** 2022-10-23

**Authors:** Joana F. A. Valente, Tiago Soares Carreira, Juliana R. Dias, Fani Sousa, Nuno Alves

**Affiliations:** 1 CDRSP-PL—Centre for Rapid and Sustainable Product Development, Polytechnic of Leiria, 2411-901 Leiria, Portugal; 2 CICS-UBI—Health Science Research Centre, University of Beira Interior, Av. Infante D. Henrique, 6200-506 Covilha, Portugal

**Keywords:** additive manufacturing, arginine, chromatography, chromatographic supports, fused deposition modelling, 3D printing

## Abstract

The increasing progression of biopharmaceutical-based therapies highlights the demand for efficient chromatographic methods that can be used to purify the desired biomolecules (e.g., nucleic acids, enzymes, or monoclonal antibodies) which are presently under consideration in clinical trials or approved by the Food and Drug Administration. These molecules present distinct chemical and structural properties, which are critical cues for the development and production of adequate chromatographic supports. Until now, it has not been possible to fully control the characteristics of the chromatographic matrices to assure the total reproducibility of their structure and packing. Meanwhile, three-dimensional printing (3DP) is in the early stage of its use in the production of chromatographic supports as a fast, very precise, and reproducible methodology. Although 3DP can provide excellent performance properties to the chromatographic structures, it cannot, per se, lead to high-quality pharmaceutical products. However, the association of affinity ligands, such as amino acids, which is possible in 3DP, could enable the attainment of high-purity yields of the desired molecules. Beyond the amino acids most widely studied as chromatographic ligands, arginine has been successfully immobilized on different chromatographic supports (namely, agarose bead matrices, macroporous matrices, and monoliths) to achieve extra-pure gene therapy products. In this research, we studied the immobilization of arginine on 3DP chromatographic supports, evaluating the stability of the ligand/chromatographic support linkage under different chromatographic conditions to determine the robustness of these new prototypes. Moreover, we also applied plasmid DNA samples to these supports to observe the practical behaviour of the developed arginine 3DP chromatographic structures.

## 1. Introduction

Chromatography is a widely used technique employed to separate and assess the chemical purity of drug substances, metabolites, and biological products in the pharmaceutical, food, and environmental fields, possessing great importance in quality control laboratories. The available chromatographic supports are based on microparticulate or monolithic structures, in which it is difficult to accomplish an ordered packaging. This may lead to deleterious effects on the chromatographic run efficiency. In general, this inability to strictly control the morphology and porosity of traditionally manufactured materials can result in low levels of reproducibility, with the frequent need to prepare and validate each column individually [1]. Additionally, since the performance of chromatographic separations depends on several factors, including the flow of the mobile phase within the column, with its influences on the axial dispersion and peak widening (resulting in low degrees of purity), scientific studies have shown that the use of ordered media provides a significantly improved chromatographic performance [2]. 

To solve this problem, additive manufacturing (AM) can be applied as a fast, highly accurate, and reproducible methodology for producing 3DP chromatographic structures. AM enables the effective control of the size, shape, position, alignment, and configuration of the structure to create complex structures that are impossible to produce conventionally [3]. The use of AM is expected to improve the performance of the separation, resolution, productivity, and cost-efficiency of chromatographic supports. 

Included among a wide range of AM techniques is fused deposition modelling (FDM), which is a process based on the extrusion of a thermopolymer through a printing nozzle to produce a layer-by-layer construction. This printing methodology has already been successfully applied by other researchers for the printing of chromatographic structures. For example, Abdulhussain et al. (2020) used different polypropylenes to print chromatographic structures through FDM, which were used for the separation of different small molecules [4]. 

Although this potential to provide the structural organization and, consequently, reproducibility of the chromatographic supports, it is well known that the coupling of specific ligands with these structures is crucial for achieving extra-pure biological products, which is a basic requirement needed to fulfil the regulatory agencies’ criteria [5]. Based on this, several affinity ligands, namely amino acids, have been successfully applied to the purification of biomolecules by chromatography. This kind of ligand can mimic the natural interaction phenomena that occur between nucleic acids and proteins in biological environments. Until now, amino acids such as arginine, histidine, and methionine, among others, have been successfully applied for the isolation of the biologically active supercoiled (sc) pDNA isoform [6]. 

In this work, we applied a new approach to produce reproducible and architecturally controlled chromatographic supports (which is not possible in the case of conventionally produced chromatographic supports). This new approach is based on additive manufacturing (AM) technology-3D printing. Additionally, arginine was immobilized in these 3D-printed structures that were then evaluated to assess the effective linkage between the amino acid and the chromatographic structure by SEM, FTIR, and EDX. Moreover, additional experiments were performed to determine whether the arginine attached to the scaffolds would disconnect when it came into contact with different buffers to assess the stability and robustness of the structures. Finally, pDNA adsorption experiments were conducted to explore the capacity of these novel chromatographic matrices to interact with the given biomolecules.

## 2. Materials and Methods

### 2.1. Materials

For the preparation of the solutions, ultrapure-grade water purified with a Milli-Q system from Millipore (Billerica, MA, USA) was used. L-Arginine and epichlorohydrin were obtained from Biochem Chemopharma (Cosne-Cours-sur-Loire, France). Sodium chloride (NaCl) and tris(hydroxymethyl) aminomethane (Tris) were obtained from Scharlab (Barcelona, Spain), sodium hydroxide (NaOH; pellets) was obtained from Fisher Chemical (Geel, Belgium), and sodium hydrogen carbonate (NaHCO_3_) was obtained from Panreac Química SLU (Barcelona, Spain). Moreover, poly (ε-caprolactone) with Mw 50.000 (CAPA 6500) in the form of 3 mm pellets was obtained from Perstorp Caprolactones (Cheshire, UK). The p53-encoding plasmid (pcDNA3–FLAG–p53) used in this work was acquired from Addgene (Cambridge, MA, USA—plasmid 10838) [1,2,3]. The NZYMaxiprep kit and the DNA ladder (Ladder III) were purchased from NZYTech (Lisbon, Portugal). 

### 2.2. Methods

#### 2.2.1. Production of the Chromatographic Scaffolds

The chromatographic scaffolds used in this work were produced using a BioExtruder device, developed at the Polytechnic of Leiria (P.L., Pt). This equipment enables the layer-by-layer extrusion of the molten PCL through a nozzle (23 gauge), with a 0/90° laydown pattern previously designed by a computer. Therefore, cylindric chromatographic structures with a 10 mm diameter, 2.6 mm height, and an interconnected porous network of square channels were obtained.

#### 2.2.2. Immobilization of Arginine in the Chromatographic Scaffolds

The arginine immobilization in the 3D-printed chromatographic structures was performed using three distinct steps. First, each scaffold was placed in a small glass vial, to which we added 3M NaOH, and then the vials were incubated at 37 °C, 100 rpm, for 5 h. Then, the NaOH was removed and the scaffolds were washed with Milli-Q H_2_O before the addition of 17 µL of epichlorohydrin, followed by incubation at 37 °C, 100 rpm overnight. In the third and final step, the epichlorohydrin was removed through washing with Milli-Q H_2_O, and then an arginine-saturated solution prepared in 2 M NaHCO_3_ was added to each vial and left incubating at 37 °C, 100 rpm, for approximately 72 h. Note that the scaffolds were completely covered by the solutions in the vials. Afterward, the arginine-saturated solution was washed with Milli-Q H_2_O, and the scaffolds were stored in 20% ethanol until further use. After the 72 h incubation period, samples of the initial arginine-saturated solution from each vial were obtained for OD_235 nm_ measurements using a spectrophotometer (BMG LABTECH, SPECTROstarNano, Offenburg, Germany) to evaluate the amount of arginine immobilized.

#### 2.2.3. Characterization of the Arginine-Modified 3DP Chromatographic Structures

To confirm whether the arginine immobilization in the scaffolds had been successful, different techniques were used. Non-immobilized scaffolds were used as the blank in all the techniques, and an *n* = 4, at the least, was considered.

##### Fourier Transform Infrared Spectroscopy (FTIR)

To progress from the immobilization process and, ultimately, identify evidence of the successful immobilization of the arginine, an FTIR analysis was performed. Briefly, a piece of the scaffolds was mounted on a diamond window and compressed to improve the spectrum signal-to-noise ratio. For each sample, 128 scans were acquired within a spectral width ranging from 4000 to 500 cm^−1^ and a spectral resolution of 4 cm^−1^ [7]. The non-immobilized matrix was used as the blank. The spectra were recorded using an FTIR spectrophotometer (Bruker, Alpha P, Bremen, Germany).

##### Scanning Electron Microscopy (SEM) and Energy-Dispersive X-ray Analysis (EDX)

SEM (Vega3 Tescan equipment from Tescan, Brno, Czech Republic)) was used to evaluate any possible morphological changes that occurred during the arginine immobilization process. For this test, samples of the scaffolds were analyzed and compared under 15 kV and at variable magnifications. The samples were attached to a brass stub with double-sided tape and coated with a gold/palladium (Au/Pd) thin film by sputtering, using the sputter coater equipment (Quorum Technologies).

Additionally, elemental analysis was performed on both the immobilized and non-immobilized scaffolds to confirm whether nitrogen (N) was present, as this can indicate the presence of arginine in the samples. To achieve the variation in the N atoms resulting from the arginine immobilization process, the samples were placed on aluminium stub supports and air-dried at room temperature, and then an EDX analysis (Bruker, XFlash Detector 6|30 from Karlsruhe, Germany) was performed. 

##### Micro-Computed Tomography Analysis (µ-CT)

The extruded scaffolds were scanned using a Skyscan 1174 (Brucker, Karlsruhe, Germany) with an image pixel size of 30.11 µm, exposure time of 7000 milliseconds, and a rotation step of 1°. The three-dimensional reconstructions were obtained using CTan and CTvox, while the transversal plane views were obtained using a data viewer. Porosity measurements (*n* = 3) were derived from the µ-CT reconstructions. 

#### 2.2.4. Evaluation of Arginine Leakage under Different Conditions

After proving the success of the arginine immobilization process, some experiments were carried out to ensure that the arginine was not easily released and washed out from the scaffolds, a process usually known as ligand leakage. Batch experiments using different buffers were thus performed to assess the stability of the immobilization. The selection of the buffers relied on the conditions that are commonly used for arginine chromatographic supports in the purification of nucleic acids, including the conditions required for support regeneration (0.1 M NaOH, used in regeneration/sanitization protocols; 2 M NaCl and 10 mM Tris-HCl, pH 8, used in the chromatographic runs; 20% ethanol, used in the storage of the scaffolds; water, used to clean the chromatographic support). For the experiments, each scaffold was placed in a glass vial, to which 1 mL of the selected buffer was added. The vials were incubated at room temperature at 100 rpm for 30 min. After incubation, the samples were analyzed by measuring the OD_235nm_ to evaluate whether the arginine was desorbed from the scaffolds. A comparison with non-functionalized chromatographic supports in contact with the same buffers was also performed. 

#### 2.2.5. Plasmid DNA Production and Isolation

The amplification of pcDNA3–FLAG–p53 was achieved using cell cultures of *E. coli* DH5α. The bacteria cultures were developed at 37 °C, 250 rpm in Erlenmeyer flasks with 500 mL of Terrific Broth medium (20 g/L of tryptone, 24 g/L of yeast extract, 4 mL/L of glycerol, 0.017 M KH_2_PO_4_, 0.072 M K_2_HPO_4_) supplemented with 30 µg/mL ampicillin. Then, *E. coli* cells were grown until the late log phase (OD_600nm_ ± 9) and then collected by centrifugation at 4000 rpm and stored at −20 °C until further use.

To recover the produced pDNA, the *E. coli* cells were first disrupted by alkaline lysis, and the p53-encoding pDNA (sc and oc isoforms) was pre-purified using the NZYTech kit according to the manufacturer’s instructions. Briefly, after the alkaline lysis, the pDNA molecules were bound to the NZYTech anion exchange resin under the appropriate low-salt and pH conditions. Then, the impurities were removed by a medium salt wash and, finally, the pDNA was eluted through the increase in ionic strength.

##### The pDNA Batch Adsorption Experiments

For the experiments, performed in batch conditions, each scaffold was placed in a glass vial, to which we added 2 mL of ((40) µg/mL) pDNA, and then the vials were incubated at 20 °C at 100 rpm for 15 min. Then, the pDNA samples were collected, and the vials were quickly washed with 1 mL of 10 mM Tris-HCl (pH 8). After the washing step, 2 mL of 2 M NaCl (pH 8) was added to the vials to induce the elution of the retained pDNA in the scaffold, followed by incubation at 20 °C at 100 rpm for 15 min. After incubation, the samples were collected. To evaluate the yield of recovered pDNA, OD_260nm_ measurements of the collected samples (in the various steps) were performed and compared with the initial pDNA solution, discounting the blank 10 mM Tris-HCl and 2 M NaCl buffers. This assay was performed in four independent assays on 4 different days. 

##### Agarose Gel Electrophoresis

After assessing the binding profile, agarose gel electrophoresis was performed to verify whether pDNA was bound to, and recovered from, the chromatographic scaffolds. Thus, a 0.7% agarose gel stained with GreenSafe dye (NZYTech, Lisbon, Portugal) was prepared in 1X TAE buffer. Before loading the samples onto the gel, the samples recovered in the elution step (with NaCl) were subjected to a concentration process (Sartorius, Gottingen, Germany) to enable better visualization of the gel. A DNA ladder (ladder III, NZYTech) and the samples (16 µL of sample + 4 µL of loading buffer (3 mL of 30% glycerol, 25 mg of bromophenol blue (0.25%, PanReac AppliChem, ITW Reagents, Chicago, IL, USA), and Milli-Q H_2_O until it reached 10 mL)) were loaded onto the gel. Finally, electrophoresis was run with 1X TAE buffer (20 mM acetic acid, 1 mM EDTA, and 40 mM Tris base, pH 8.0) at 100 V for around 40 min, and the resulting gel was observed under UV light in a transilluminator (SmartDoc™, Accuris Instruments, Edison, NJ, USA).

## 3. Results and Discussion

The AM method used to produce the chromatographic structures was FDM. This is an economic and easy-to-use 3DP methodology with the ability to create structures of various shapes, with a low-price instrument, no need for chemical post-processing, no resins to cure, and a broad range of thermoplastics that can be successfully applied to the 3DP pieces [4]. In this work, the polymer chosen for the printing of the chromatographic structure was PCL, a material that is very easy to handle, with a slightly low fusion temperature (around 60 °C) that enables the achievement of robust pieces [8]. This is an important parameter to consider since due to the flow rate or the type of sample used, the pressure achieved during the chromatographic run can reach several “psi”, leading to chromatographic structure damage [9]. 

### 3.1. Chemical Evaluation of the Immobilization Process of Arginine

One of the critical aspects of chromatographic support production is the effective immobilization of the desirable ligands. In this process, one must assure the stable linkage of the ligands to the chromatographic structure and its modification with a large number of ligands to reach the highest ligand density possible [10]. These two features significantly contribute to the support’s robustness and increased binding capacity, which is very important for the purification results.

**Figure 1:**
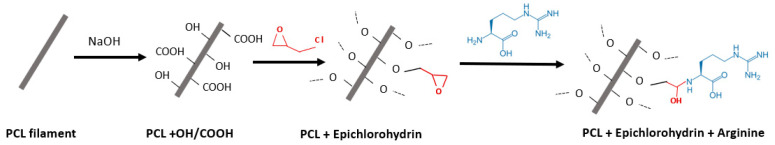
Schematic representation of the chemical arrangements produced during the arginine’s immobilization on the 3D-Printed PCL structure (based on Valente et al. 2020 and Pourrostam-Ravadanaq et al., 2020 [12,13]).

In this work, the immobilization process included three key steps, with the first comprising the PCL scaffold’s immersion in NaOH, leading to the formation of hydroxyl (OH) and carboxylic (COOH) groups on the PCL filament surface; the second step comprising the use of epichlorohydrin to enrich the structure with epoxy groups (binding to the previously added -OH groups); and the third and final step is responsible for the linkage of the arginine to the 3D-printed chromatographic structure (Figure 1) [11].

The surface of the PCL-printed chromatographic structures was firstly soaped in NaOH to obtain anchorage zones for the epoxy ring. Then, the PCL surface was activated using epichlorohydrin through a nucleophilic substitution reaction. Finally, the L-arginine ligand was attached to the surface of the activated PCL structure by its amine group through the ring-opening base reaction [13]. 

**Figure 2:**
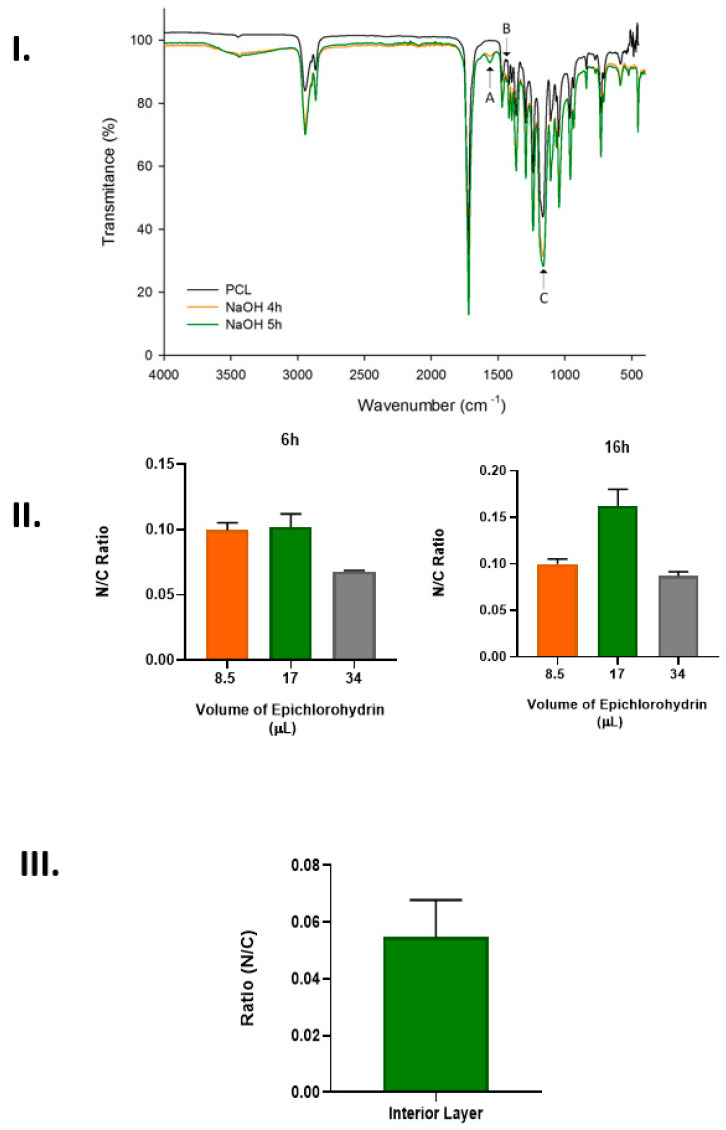
Assessment of the arginine’s immobilization on the 3D-printed chromatographic support. (**I**) FTIR analysis of the 3D-printed PCL after 4 h and 5 h of contact with 3 M of NaOH—A and B could represent ester bond cleavage, demonstrating the PCL modification and C represents the peak with where can be visualized the available anchoring sites for epoxy group bonding; (**II**) EDX analysis of the PCL surface exposed to different amounts of epichlorohydrin for 6 and 16 h; (**III**) EDX analysis of the internal layer of the 3D-Printed chromatographic structure after the immobilization process (*n* = 4).

Having completed the immobilization procedure, it was necessary to verify that the arginine was efficiently connected to the chromatographic support and, to achieve this, different techniques, including FTIR, EDX, and UV spectroscopy, were performed (Figure 2). 

In Figure 2I, it is possible to observe the FTIR spectrum of the PCL structure before and after it came into contact with 3 M of NaOH for 4 and 5 h. Representative peaks of the PCL were observed at 2939 cm^−1^ (asymmetric CH_2_ stretching), 2865 cm^−1^ (symmetric CH_2_), 1718 cm^−1^ (carbonyl C=O stretching), 1188 cm^−1^ (C-O-H stretching), and 1163 cm^−1^ (C-O-C stretching) [14]. The peaks I–A (1567 cm^−1^) and B (1431 cm^−1^), which are presented in the samples within 4 h and 5 h of incubation with NaOH, can be related to the ester bond cleavage, demonstrating the PCL modification. Moreover, the sample modified during 5 h presented a higher peak intensity than the 4 h modified samples due to the higher modification level. Additionally, Figure 2IC shows an increase in the intensity of the C-O-H (carboxyl and hydroxyl) peak (1188 cm^−1^) compared to the intensity of the C-O-C (ester) peak (1163 cm^−1^) [15]. This intensity increase was more significant in the 5 h NaOH-treated than the 4 h NaOH-treated chromatographic supports due to the higher modification, which represents a larger number of available anchoring sites for epoxy group bonding. 

Regarding the second step of the immobilization process, which is represented by the results presented in Figure 2II, it is possible to observe that both studied factors, namely, the quantity of epichlorohydrin and the contact time, influenced the efficiency of the epoxy groups’ coupling to the PCL structure. The results show the number of nitrogen atoms (from the arginine) on the PCL structures incubated with different amounts of epichlorohydrin for 6 and 16 h. The data suggest that 16 h of contact with epichlorohydrin was more efficient in ensuring arginine coupling to the structure, while 17 µL was the ideal amount of this reagent required to promote greater arginine immobilization. However, even higher amounts of epichlorohydrin or increased contact times would not be beneficial for the immobilization process due to the corrosive properties of the reagent, which could degrade the PCL surface. The last step of the immobilization process comprised the binding of the arginine to the epoxy-activated chromatographic structure. 

In Figure 2III, it is possible to observe the results obtained after the assessment of the N atoms of the arginine ligand in the internal region of the PCL chromatographic structure. It was observed that arginine linkage occurred in all the structures. However, on the surface, the amount of N atoms and, consequently, the amount of arginine were higher in comparison to the amount of these atoms present in the interior of the structure. This could be due to the porous dimension, which can lead to difficulty regarding the arginine ligands’ ability to access the internal structure. To overcome this limitation, different immobilization conditions were used, specifically by increasing the contact time with the different immobilization solutions and increasing the mixing velocity during the process. 

Comparing these results with others, the percentage of N atoms seemed to be diminished in our 3D-printed matrices [12]. However, it is important to note that this previously cited study does not refer to the ratio between the N atom and residual atoms, such as carbon, for example. This is an important step that must be performed since one can measure more dense spots, leading to an up-measurement of the N atoms. 

### 3.2. Morphological Evaluation of the 3D-Printed Structures

#### SEM

**Figure 3:**
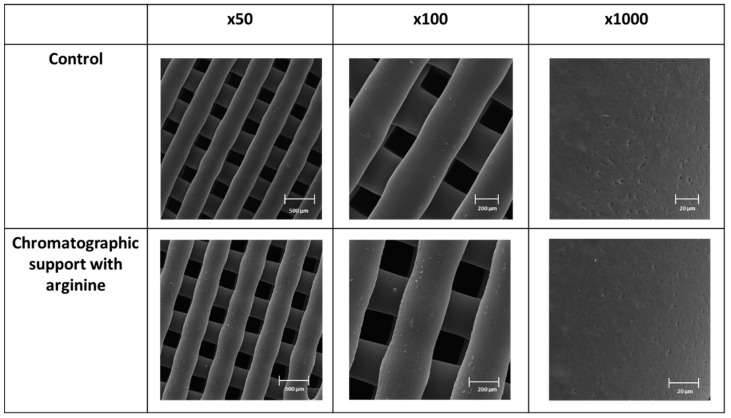
SEM analysis (with magnifications of 50, 100, and 1000×) of the 3D-printed PCL structure before and after the immobilization process.

**Figure 4:**
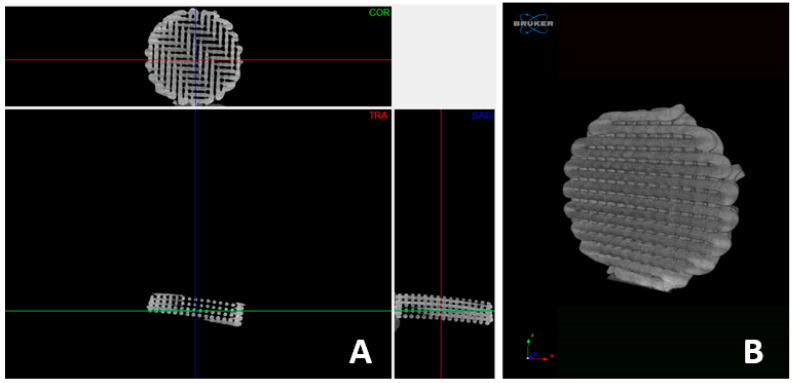
The 3D projections: Micro-CT generated 3-dimensional projections: (**A**) transversal plane views; (**B**) micro-computed tomography (µ-CT) 3D reconstruction of a representative extruded PCL chromatographic structure.

An in-depth analysis of the 3DP PCL structures before and after the immobilization process was also performed through SEM (Figure 3) and µ-CT (Figure 4). This characterization was performed to evaluate the morphology, structure, piece robustness, filament alignment, and pore assessment and to guarantee the maintenance of the integrity/stability of the printed structures after the immobilization process.

The SEM analysis (Figure 3) showed that, although the immobilization process could aggressively impact the printed structure, no degradation was observed, independently of the ampliations used in this analysis. This may indicate that the experimental conditions used for the arginine immobilization are appropriate and do not damage the chromatographic support. This is extremely important since the degradation of the surface can be an issue affecting the lifetime of the chromatographic structures in general [16].

Through the analysis performed in this study, it was also possible to measure the diameter of the filaments, which was around 342.2 ± 14 µm, a slightly higher value compared with the diameter of the extrusion needle. Moreover, the dimensions of the pores were also assessed, reaching an average value of 274 ± 4 µm. This porosity enabled the buffers and samples to flow through the internal and external structure of the 3DP chromatographic structure. These dimensions can lead the 3D-printed structures to acquire some of the advantages of the macroporous conventional supports, such as the ability to improve the access of pDNA to the internal voids and to enable the convective pore flow, consequently improving the internal mass transfer [12,17]. 

When compared with other types of chromatographic structures, such as the microparticulated structures, the beads can range from 30 to 500 µm and the pore size can range from 30 µm to 20 nm. Concerning the monolithic supports, the fiber usually ranges from 0.7 to 2 µm and the pore size average is 4.5 µm. Among these parameters, one of the most important to consider when designing the chromatographic piece is the pore dimensions, since the average pore size needs to be at least the same size as the radius of gyration of a pDNA molecule [18]. 

### 3.3. µ-CT

To better assess the internal morphology of the 3DP structure and achieve the exact values of the porosity and percentage of the pores, µ-CT was used (Figure 4). This technique is extremely valuable because it helps researchers to achieve the parameters previously described without cutting or manipulating the studied samples.

Through the analysis of these projections, it is possible to observe that the 3D-printed support comprises interconnected channels that lead to an interconnected structure, which is a key point for this kind of 3D structure that must be applied in the chromatographic field. Additionally, through the acquired projections, it was possible to determine the structure’s porosity as around 87% ± 4.3 (*n* = 3). 

Comparing these results with the conventionally used/produced chromatographic structures, it is possible to observe that the obtained values are higher than the range of the values produced up to now. Therefore, in a microparticulate chromatographic structure, the external porosity is typically 40%, with values ranging between 37% and 42%. The porosity of the macropore network, which, in monolithic columns, plays the same role as that which the external or interparticle pore network plays in packed columns, is typically around 70% [18].

### 3.4. Assessment of the Immobilization Process Quality

**Figure 5:**
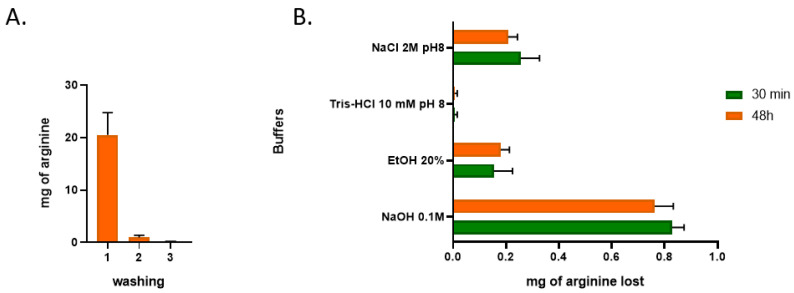
Loss of arginine after the 3D-printed chromatographic structures came into contact with different buffers. (**A**) Arginine lost after several washing steps with water; (**B**) arginine lost in PCL structures washed twice with water, after coming into contact with different buffers for 30 min or 48 h (*n* = 3).

Guaranteeing the effective linkage of the ligand to the chromatographic support is extremely important for the chromatographic process, as it determines the efficiency of the target biomolecules’ retention, also influencing the maximum binding capacity of the support, with an impact on the recovery yield. In this regard, when we have a solid 3D structure, it is important to assure that the immobilization process is efficient not only on the surface of the piece but also on the internal part of the structure, which, due to the geometry of the 3D-printed structure, is usually more difficult to access. In this regard, in Figure 5, it is possible to observe the loss of arginine after the 3D-printed structures came into contact with different buffers.

In Figure 5, it is possible to observe that the first washing step led to a loss of 20 mg of the ligand, which could be due to the washing of free arginine that was not fully linked to the PCL structure. It is also possible to observe that, in the following washing steps, only residual losses of this ligand could be verified, being extremely favourable for the chromatographic process since, at least in theory, it can promote reproducible results over the chromatographic support’s lifetime. Therefore, we can conclude that nearly 70% of the initially calculated amount of arginine immobilized was still immobilized on the chromatographic piece. 

Another important piece of information that can be observed from the analysis of Figure 5 is related to the use of different buffers in this 3DP chromatographic matrix immobilized with arginine ligands. From these results, it is possible to observe that the loss of ligands is residual/null in the majority of the cases. The only buffer that leads to a slight increase in the arginine loss is NaOH, when it is applied. NaOH is usually applied to promote a deeper cleaning of the chromatographic structures, mainly to guarantee their reuse [19]. However, using NaOH can damage the ligand over repeated cycling, leading to a decrease in the dynamic binding capacity, which is why the chromatographic resin suppliers recommend using this buffer only when extensive regeneration is needed [20,21]. In any case, concerning the initial amount of amino acid retained on the 3DP PCL structure, each cycle of NaOH leads to an arginine loss of 1.1 %, which, in theory, means that 24 NaOH cycles are needed for a 50% loss in the arginine ligands’ linkage to the PCL support.

### 3.5. The pDNA Adsorption Ability

Arginine was selected for immobilization in the 3D-printed chromatographic supports mainly because it has already been successfully applied in the past for the selective recovery of different biomolecules and types of supports [12,22,23,24]. Therefore, it is crucial to evaluate the chromatographic support’s ability to interact with the target sample during the support development process. 

**Figure 6:**
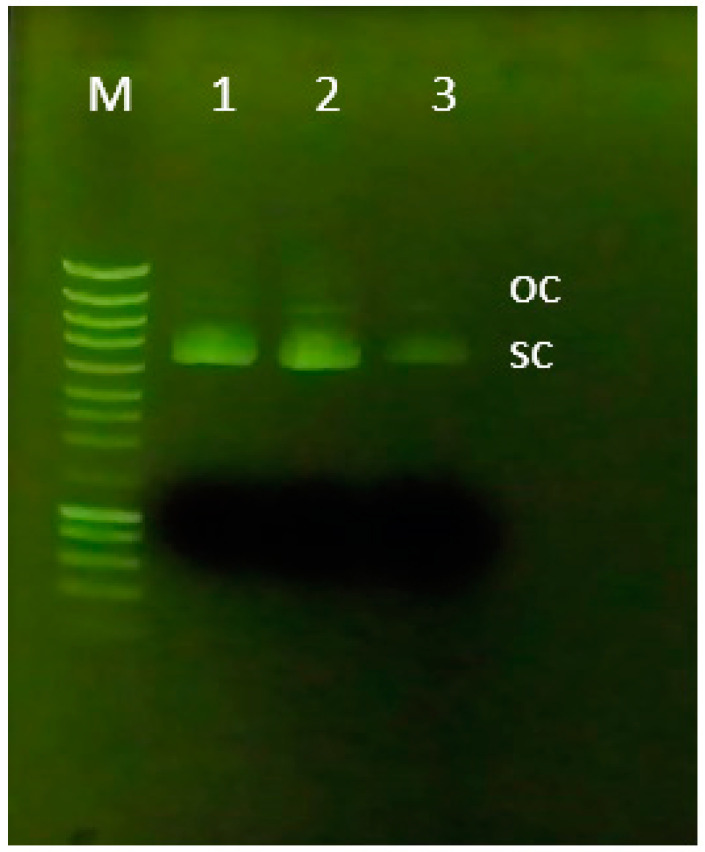
Analysis of the agarose gel electrophoresis of samples collected from each peak in the purification assay. Lane M: molecular weight marker; Lane 1: feed sample (*E. coli* lysate pre-purified with a commercial kit); Lane 2: pDNA + Tris 10 mM pH 8 chromatographic support; Lane 3: pDNA + NaCl 2M pH 8 chromatographic support.

Concerning this, the developed chromatographic support was placed in contact with a pDNA pre-purified sample (sc + oc isoforms), and the ability to interact with this biomolecule was evaluated. Since the arginine ligand has already been described as an affinity ligand that can promote multiple non-covalent interactions, it is possible to modulate the performed interactions with the target sample by manipulating the chromatographic conditions, namely the salt used. In this work, we decided to use NaCl to exploit mainly ionic interactions (since this kind of interaction between arginine and pDNA samples has provided good results [12,25]). The agarose analysis of this experiment is presented in Figure 6 where is possible to observe the profile of each peack of the purification assay. Additionally, from a complementary analysis was also possible to measurea total of 0.6 µg/mm^3^ pDNA adsorbed on the 3D-printed chromatographic support.

Therefore, it is worth mentioning that the arginine ligand enables the establishment of a multitude of interactions with the sc pDNA, such as hydrophobic, electrostatic, and cation-π interactions, van der Waals forces, and/or hydrogen bonds. Van der Waals forces and water-mediated bonds can contribute to the amino acid–DNA interactions and studies have shown that the arginine specificity is mainly due to hydrogen bonds, which are favoured by bidentate and complex interactions, preferentially with the guanine base [26].

Considering the obtained results, it is suggested that a larger chromatographic piece is needed for an increased level of pDNA binding. However, it is also relevant to mention that the great porosity and the contribution of the macropores can provide these 3D-printed chromatographic structures with higher permeability and higher efficiency than those of the conventional columns. Moreover, it is possible to use more dense extracts without compromising the flow within the 3D-printed chromatographic structure and, additionally, it is possible to operate these matrices at higher velocities to carry out faster analyses. 

## 4. Conclusions

As mentioned before, chromatographic approaches must be improved in such a way that the chromatographic bed can be fully organized to guarantee its reproducibility and enable the prediction and the full computational simulation of the chromatographic process. To achieve this, and to create exceptional chromatographic models that can overcome the existent models on the market, a mutual effort between scientists and industry is needed.

Therefore, 3D printing has a panoply of applications, and its use to produce chromatographic structures remains little explored. In light of this, in this research work, we presented the production and development of a 3D-printed chromatographic structure designed to be applied in affinity chromatography.

We demonstrated the successful production of the PCL matrix using FDM, and then arginine ligands were successfully immobilized on its surface. Several experiments were performed to guarantee the stability of the linkage between the chromatographic support and arginine using complementary techniques and different buffers (the buffers were chosen taking into account those that are commonly applied in chromatographic experiments and the sanitation of a conventional arginine chromatographic support). The interaction between the ligand and the pDNA was also evaluated, and the 3D-printed chromatographic support was able to interact with the target sample. Overall, it was possible to produce arginine chromatographic supports that can be applied as pDNA adsorbers, with a fully controlled geometry, using 3D printing methodologies.

Concerning the developed structures, it is also interesting to note that the large pores enable the application of more dense extracts, without flogging or clogging, which creates the possibility of using these structures to recover different biomolecules/drugs. Moreover, with the achieved architecture and its easy manipulation/production ability, it may be possible to use these new supports for substance/biomolecule quantification. Additionally, since arginine has been used as a specific ligand, it is possible to guarantee that the 3D-printed structure will enable the separation of other kinds of biomolecules [24]. Finally, since the developed 3D-printed support was successfully activated via epoxy, it can enable the immobilization of other ligands, exponentially increasing the range of application of the developed structure.

## Data Availability

Not applicable.

## References

[1] Schure M.R., Maier R.S., Kroll D.M., Davis H.T. (2004). Simulation of ordered packed beds in chromatography. J. Chromatogr. A.

[2] Kalsoom U., Nesterenko P.N., Paull B. (2018). Current and future impact of 3D printing on the separation sciences. TrAC Trends Anal. Chem..

[3] Valente J.F.A., Sousa F., Alves N. (2021). Additive Manufacturing Tools to Improve the Performance of Chromatographic Approaches. Trends Biotechnol..

[4] Abdulhussain N., Nawada S., Currivan S., Passamonti M., Schoenmakers P. (2020). Fabrication of polymer monoliths within the confines of non-transparent 3D-printed polymer housings. J. Chromatogr. A.

[5] Sousa A., Almeida A.M., Valente J., Queiroz J.o., Sousa F. (2022). Hands-On Laboratory Class for Biopharmaceutical pDNA Quality Control. J. Chem. Educ..

[6] Carapito R., Valente J.F.A., Queiroz J.A., Sousa F. (2022). Arginine-Affinity Chromatography for Nucleic Acid (DNA and RNA) Isolation. Affinity Chromatography.

[7] Fuji T., Anada T., Honda Y., Shiwaku Y., Koike H., Kamakura S., Sasaki K., Suzuki O. (2009). Octacalcium phosphate–precipitated alginate scaffold for bone regeneration. Tissue Eng. Part A.

[8] Domingos M., Chiellini F., Cometa S., De Giglio E., Grillo-Fernandes E., Bártolo P., Chiellini E. (2010). Evaluation of in vitro degradation of PCL scaffolds fabricated via BioExtrusion. Part 1: Influence of the degradation environment. Virtual Phys. Prototyp..

[9] Pfaunmiller E.L., Paulemond M.L., Dupper C.M., Hage D.S. (2013). Affinity monolith chromatography: A review of principles and recent analytical applications. Anal. Bioanal. Chem..

[10] Valente J.F.A., Queiroz J.A., Sousa F. (2021). Dilemma on plasmid DNA purification: Binding capacity vs selectivity. J. Chromatogr. A.

[11] Yew C.H.T., Azari P., Choi J.R., Muhamad F., Pingguan-Murphy B. (2018). Electrospun polycaprolactone nanofibers as a reaction membrane for lateral flow assay. Polymers.

[12] Valente J.F.A., Sousa A., Azevedo G., Queiroz J., Sousa F. (2020). Purification of supercoiled p53-encoding plasmid using an arginine-modified macroporous support. J. Chromatogr. A.

[13] Pourrostam-Ravadanaq P., Safa K.D., Abbasi H. (2020). Study of imidazole performance as pseudo-affinity ligand in the purification of IgG from bovine milk. Anal. Biochem..

[14] Dias J.R., Dos Santos C., Horta J., Granja P.L., Bártolo P.J. (2017). A new design of an electrospinning apparatus for tissue engineering applications. Int. J. Bioprinting.

[15] Zamani Y., Mohammadi J., Amoabediny G., Visscher D.O., Helder M.N., Zandieh-Doulabi B., Klein-Nulend J. (2018). Enhanced osteogenic activity by MC3T3-E1 pre-osteoblasts on chemically surface-modified poly (ε-caprolactone) 3D-printed scaffolds compared to RGD immobilized scaffolds. Biomed. Mater..

[16] Vidič J., Podgornik A., Jančar J., Frankovič V., Košir B., Lendero N., Čuček K., Krajnc M., Štrancar A. (2007). Chemical and chromatographic stability of methacrylate-based monolithic columns. J. Chromatogr. A.

[17] Deshmukh N., Lali A. (2005). Adsorptive purification of pDNA on superporous rigid cross-linked cellulose matrix. J. Chromatogr. B.

[18] Al-Bokari M., Cherrak D., Guiochon G. (2002). Determination of the porosities of monolithic columns by inverse size-exclusion chromatography. J. Chromatogr. A.

[19] Yang L., Harding J.D., Ivanov A.V., Ramasubramanyan N., Dong D.D. (2015). Effect of cleaning agents and additives on Protein A ligand degradation and chromatography performance. J. Chromatogr. A.

[20] Linhult M., Gülich S., Gräslund T., Simon A., Karlsson M., Sjöberg A., Nord K., Hober S. (2004). Improving the tolerance of a protein a analogue to repeated alkaline exposures using a bypass mutagenesis approach. Proteins Struct. Funct. Bioinform..

[21] Hober S., Nord K., Linhult M. (2007). Protein A chromatography for antibody purification. J. Chromatogr. B.

[22] Soares A., Queiroz J.A., Sousa F., Sousa A. (2013). Purification of human papillomavirus 16 E6/E7 plasmid deoxyribonucleic acid-based vaccine using an arginine modified monolithic support. J. Chromatogr. A.

[23] Cardoso S., de Alcântara Pessoa Filho P., Sousa F., Azzoni A.R. (2018). Arginine and di-arginine ligands for plasmid DNA purification using negative chromatography. Sep. Purif. Technol..

[24] Martins R., Queiroz J., Sousa F. (2013). New approach in RNA quantification using arginine-affinity chromatography: Potential application in eukaryotic and chemically synthesized RNA. Anal. Bioanal. Chem..

[25] Sousa F., Matos T., Prazeres D., Queiroz J.A. (2008). Specific recognition of supercoiled plasmid DNA in arginine affinity chromatography. Anal. Biochem..

[26] Luscombe N.M., Laskowski R.A., Thornton J.M. (2001). Amino acid–base interactions: A three-dimensional analysis of protein–DNA interactions at an atomic level. Nucleic Acids Res..

